# Understanding the Food and Nutrition Insecurity Drivers in Some Emergency-Affected Countries in the Eastern Mediterranean Region from 2020 to 2024

**DOI:** 10.3390/nu16223853

**Published:** 2024-11-11

**Authors:** Salwa Jamal Al Sharjabi, Ayoub Al Jawaldeh, Ola El Hajj Hassan, Fekri Dureab

**Affiliations:** 1 Regional Office for the Eastern Mediterranean, World Health Organization (WHO), Cairo 7608, Egypt; aljawaldeha@who.int; 2 Heidelberg Institute of Global Health, Hospital University Heidelberg, 69120 Heidelberg, Germany; ola.elhajjhassan@uni-heidelberg.de (O.E.H.H.); dureabf@who.int (F.D.)

**Keywords:** food security, malnutrition, children, emergency, conflicts

## Abstract

This research seeks to enhance the understanding of the multifaceted drivers of food and nutrition insecurity in emergency-affected countries within the Eastern Mediterranean region and investigate the dynamics of food and nutrition security in countries facing emerging emergencies. This is a descriptive aim to determine the key factors and challenges affecting food security and nutrition status in ten countries in the Eastern Mediterranean region (Afghanistan, Djibouti, Iraq, Lebanon, Pakistan, Palestine (Gaza Strip), Somalia, Sudan, Syria, and Yemen). The research reveals that all selected countries experienced severe levels of food insecurity, with many reaching Phase 3 or above according to the IPC classification. In 2020, Afghanistan and Yemen were particularly hard-hit, with food insecurity affecting 42% and 45% of their populations; in 2024 in Gaza and Sudan, the same figures were 93% and 54% of the population, respectively, representing worse food insecurity crises in the region. Somalia, Sudan, and Djibouti also faced significant food insecurity rates. Many key drivers of food security are standard in most countries, and the linkage between food insecurity and malnutrition levels has a similar trend in almost all countries. However, none of the countries achieved all the 2025 global nutrition targets, while some reached one or two targets. Reaching sustainable development goals is still challenging in these countries since nutrition and food security levels, included in many goals, have not yet been reached. Food security and malnutrition in emergency-affected countries are driven by conflict, political instability, natural disasters, and socioeconomic conditions, which disrupt agricultural activities and infrastructure, exacerbating these challenges. To address these issues, we recommend a multisectoral approach, conflict resolution, climate-smart agriculture, integration of emergency responses with long-term strategies, and strengthening health and nutrition information systems.

## 1. Introduction

Several countries in the Eastern Mediterranean region are currently grappling with or recuperating from emergencies, making the study of food and nutrition insecurity drivers in these contexts a crucial and timely research topic. Countries that have been facing conflict-related emergencies for many years (Afghanistan, Djibouti, Iraq, Libya, occupied Palestine, Pakistan, Somalia, Sudan, The Syrian Arab Republic, and Yemen) have been dealing with other crises and disasters. The period from 2020 to 2024 has been marked by a confluence of crises in the Eastern Mediterranean region, including ongoing conflicts, population displacement, and the additional strain caused by the COVID-19 pandemic. These emergencies could significantly impact communities’ food security and nutritional status, leading to widespread hunger, malnutrition, and long-term health consequences. Natural disasters like climate change and the spread of infectious diseases added more burden on health, food security and nutrition status, especially for the most vulnerable groups in the community: children under five years old and pregnant and lactating women [1].

Food security has four main dimensions that interact with each other. These are food availability, access, utilization, and stability. The nature and severity of the emergency could affect one or more food security dimensions, affecting other life aspects and nutrition and health status in the community [2]. “Food security exists when all people, at all times, have physical and economic access to sufficient, safe and nutritious food that meets their dietary needs and food preferences for an active and healthy life” [3].

According to the Integrated food security phase classification (IPC), the level of food insecurity has five phases based on specific criteria. The IPC Acute Food Insecurity classification differentiates between different levels of severity of acute food insecurity, classifying units of analysis into five distinct phases: minimal/none, stressed, crisis, emergency, and catastrophe/famine. Countries in this paper face food insecurity that could reach Phase 3 and above. In some conflict-related areas, food insecurity could reach the level of famine [4].

Emergencies can disrupt food systems and livelihoods, leading to increased vulnerability to malnutrition. When food availability and access are limited, malnutrition rates tend to be higher. Therefore, a higher IPC phase typically indicates a more severe nutrition situation. Malnutrition is a big challenge for the achievement of sustainable development goals. According to UNICEF’s conceptual framework, many factors could lead to malnutrition, either immediate, underlying, or fundamental; food insecurity is one of these causes [5]. Recognizing the challenges globally affecting nutrition led to the endorsement of global nutrition targets with its six pillars related to the double burden of malnutrition in the most vulnerable group in the population [6].

Malnutrition and food insecurity do not affect only the physical status of the population; they also affect the entire life aspects and development. There are strong links between nutritional status and food security, and both do not only represent sustainable development goals or targets. They are parts of human rights [7]. Countries could face different kinds of emergencies, but the impact on the population’s nutritional status could be similar. Ultimately, this research seeks to enhance the understanding of the multifaceted drivers of food and nutrition insecurity in emergency-affected countries within the Eastern Mediterranean region and investigate the dynamics of food and nutrition security in countries facing emerging emergencies, focusing on identifying key drivers of insecurity and determining the challenges in the selected countries.

## 2. Methodology

This is a descriptive aim to determine the key factors and challenges affecting food security and nutrition status in ten countries in the Eastern Mediterranean region (Afghanistan, Djibouti, Iraq, Lebanon, Pakistan, Palestine (Gaza Strip), Somalia, Sudan, Syria, Yemen).

The quantitative part of the study is collected from reports of IPC analysis (Integrated food security phase classification), Nutrition Clusters’ reports, Relief Web and WHO global nutrition targets tracking tools.

The total number of children under five in each country was collected from the population pyramid to reflect malnutrition among children under five, where rates are absent and only numbers are found in reports or collected data. In some IPC analyses for malnutrition in countries, GAM rates varied from different zones and provinces; the median was calculated in countries witnessing different GAM rates. From 2020 to the latest analysis found during the search in 2024, IPC analysis and its 5 phases were represented in the selected countries differently.

Data related to global nutrition target updates was collected from the WHO tracking tool to estimate the progress each country achieved in each goal.

Qualitative data were collected from Humanitarian Response Plans (HRPs) for the countries, IPC analysis and Humanitarian need overview (HNO) UN organization reports and Humanitarian response plans.

Keywords used for web search were IPC analysis for the period of 2020–2024 for each country, malnutrition, SAM, and GAM rates, HRP and HNO for each country, and MICS analysis for the period of 2020–2024.

### Selected Countries

**Figure 1:**
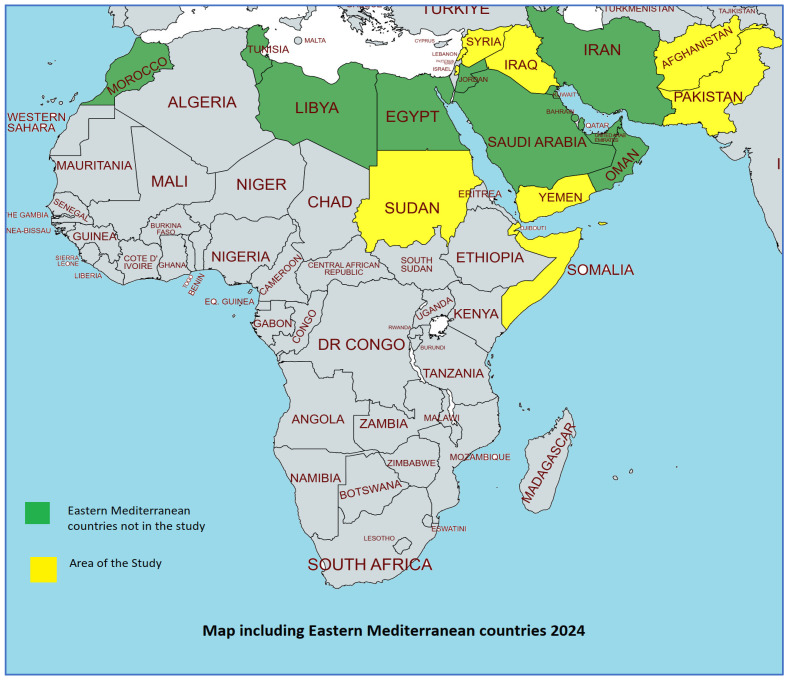
Map of the Eastern mediterranean countries [8].

Selection of 10 countries facing crisis for an extended period:

(Afghanistan, Djibouti, Iraq, Lebanon, Pakistan, Palestine, Somalia, Sudan, Syria and Yemen). The selected countries are represented Figure 1.

## 3. Results

According to the latest projection for each selected year, all the countries faced levels of food insecurity either in Phase 3 or above. The tables included in the results compare the level of food insecurity, malnutrition and the common factors affecting food security in the study areas. Table 1 represents all food insecurity phase rates in each country and each year.

In 2020, the IPC classification showed that Afghanistan and Yemen suffered from the highest rates of food insecurity compared to other countries (42%, 45%); Table 2 represents the rates of food insecurity phase above Phase 3, which is considered critical. Somalia, Sudan, and Djibouti have similar rates for food insecurity in Phase 3 and above.

In 2021, Afghanistan was still witnessing a high level of food insecurity, with 47% of households in Phase 3 and above. Yemen recorded an increase in the level of food insecurity by around 10%. Other countries registered rates like those in the previous year.

In 2022, all the countries witnessed an increase in food insecurity rates. Yemen had the highest rate (60%), Afghanistan had more than 50%, and Pakistan, Somalia, and Lebanon had rates between 37% and 42%. Sudan and Djibouti had the lowest rates compared to other countries.

Countries experienced Phases 3 and 4 of food insecurity from 2020 to 2022. The IPC analysis did not include the entirety of Palestine but only the Gaza Strip, where food insecurity in the phase of famine (Phase 5) was expected. In 2024, the Phase 5 food insecurity rate reached 50% in Gaza.

According to Table 2, in 2023, a decrease in the rates of food insecurity in Phase 3 and above was noticed in most countries except Lebanon. IPC analysis was conducted for the first time in the Gaza Strip in 2023 and reflected a high level of food insecurity, where Phases 3, 4, and 5 were registered and represented 93%.

At the end of 2023 and the beginning of 2024 in the February projection, Sudan witnessed a more than 15% increase in food insecurity; the latest projection for IPC analysis for Sudan showed a 12% increase in the food insecurity level from February to the latest projection in October 2024. Other countries faced a slight increase between 3% and 5%. Somalia was the only country that showed a decrease in food insecurity rate in 2024.

Table 3 and Table 4 Represent the number of cases and prevalence of malnutrition among children under five years in 10 countries.

Available data in 2020 shows different rates of GAM, SAM, and MAM in 10 countries where some zones and provinces exceeded the emergency threshold of malnutrition according to WHO classification. Palestine and Syria had the lowest GAM rates among the groups analyzed. GAM rates exceeded the critical threshold and were registered in Afghanistan, some provinces of Pakistan and Sudan, where serious GAM rates were found in Somalia, Djibouti, some provinces of Pakistan, and Yemen. Palestine and Syria registered poor GAM rates. The highest GAM rates in all the countries did not exceed 30% of the extreme critical value.

In 2021, data were available only for five countries. The GAM rate was the same in Afghanistan, 17%, with a slight decrease in Somalia from 13.8% to 13.1%. There was a 3.8% increase in malnutrition rates in Syria. Some zones in Yemen registered very critical GAM rates, 31%, while the median GAM rate in Yemen increased by 1% compared to the previous year. Lebanon registered an acceptable GAM rate of less than 5% in 2021.

In 2022, GAM rates in improved in Afghanistan and Somalia, while malnutrition rates continued to increase in Yemen, Syria, Djibouti, and Pakistan.

In Sudan, there was a decrease in the number of malnourished children under five from 2021 to 2022; according to Table 4, GAM cases in 2021 were around 3 million cases, while in 2022, it was about 2.76 million. Data for Iraq, Lebanon, and Palestine were missing.

GAM rates were available for only four countries in 2023. Afghanistan witnessed a significant decrease in the GAM rate from 14.1% to 3.7%. GAM rates increased in Yemen, Syria, Sudan, and Somalia.

The results in Table 5 describe the global nutrition targets for the 10 countries and the latest achievements related to six indicators.

Each country that is considered a WHO Member State has endorsed global targets for improving maternal, infant and young child nutrition and is committed to monitoring progress. Six indicators represent the globally targeted level that each country has reached: stunting, wasting, anemia, low birth weight, exclusive breastfeeding, overweight.

Regarding stunting, four countries are on track to reach the target: Iraq, Djibouti, Palestine, and Lebanon. Analysis and data related to wasting were not available for the four years except in Lebanon, while the years of analysis varied from 2009 to 2014 in other countries. Afghanistan almost reached the target in 2018, and Iraq and Pakistan are on track to achieve the targeted rate. None of the countries have reached progress in decreasing the rate of anemia among women of childbearing age.

Data related to the rate of low birth weight and reached targets were not available in almost all the countries except Palestine and Lebanon, where both countries were in the process of achieving their targeted goals.

Afghanistan was the only country reaching the target for exclusive breastfeeding. According to the latest available data, Somalia and Sudan were close to reaching the target, whereas other countries are still far from reaching the target goal. Rates related to overweight in the 10 countries show that all countries are on track to reaching their targeted rates to reduce overweight among children.

Table 6 provides a detailed overview of the main factors contributing to food insecurity and malnutrition in selected countries within the Eastern Mediterranean region (EMRO) from 2020 to 2023. The drivers are categorized by year and country, illustrating how different challenges have impacted food security over time. These include conflict, economic decline, high food prices, climate-related shocks, and limited access to essential services, all contributing to increased food insecurity and malnutrition over time. The ongoing impacts of global and regional crises, such as the COVID-19 pandemic and the Ukraine conflict, have further exacerbated these issues, underscoring the need for comprehensive and sustained interventions to address food insecurity in the region.

Conflicts and unstable political situations appeared to be among the main drivers affecting almost all the countries in the study. Afghanistan, Yemen, Sudan and the Gaza Strip are still facing current challenges related to conflicts. Prolonged conflict duration in Pakistan and Somalia affected livestock and the economic situation. In contrast, the deteriorating situation in occupied Palestine, especially in the Gaza Strip, affected food security and access to all services.

The COVID-19 pandemic is one of the main drivers of food insecurity in all countries in 2020–2021. Climate change has played a role in countries in different ways; floods in Afghanistan, Pakistan, Somalia, Djibouti, and Sudan occurred in the years 2020 and 2021. Countries that suffered from prolonged Droughts and reduced rail falls at the end of 2021 and 2022 were Somalia, Afghanistan, and Pakistan. Economic challenges such as inflation, increases in prices of services and food and reduced employment opportunities were common in all countries. Yemen and Sudan highlighted these factors during the entire period of analysis. Locust infestation affected Pakistan, Somalia, and Djibouti in 2020 and 2021. Other factors related to access to health services and the outbreak of diseases affecting people and livestock affected Somalia, Yemen, Pakistan, and Djibouti. WASH services and the availability of safe water were common factors in the countries in the analysis.

## 4. Discussion

**Figure 2:**
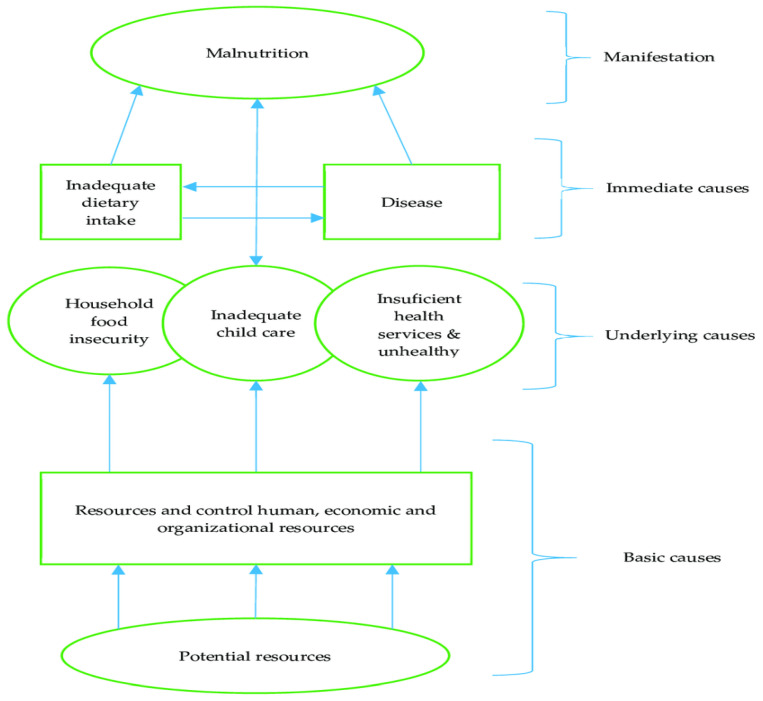
UNICEF malnutrition conceptual framework.

Malnutrition in the Eastern Mediterranean region has taken many forms throughout time. Most countries, especially those affected by conflict, experience high levels of food insecurity and undernutrition, and some countries in the paper experience either deterioration or improvement in food security or nutrition status, each according to the type of crisis and critical drivers affecting them. This study discussed the main key drivers in the countries that interact to affect food security. Those factors vary according to the area, time, and duration. According to UNICEF’s conceptual framework represented in Figure 2, food security is considered one of the underlying causes of malnutrition [104].

Key drivers influencing food security and nutritional status include both man-made and natural factors. All the countries examined in this study have endured political instability, continuous conflicts, or hostilities. For instance, Sudan has experienced prolonged political instability, which escalated in 2022. This instability, combined with a significant increase in food and commodity prices, a reduced harvest, and ongoing conflict, has led to a rapid worsening of acute food insecurity. Furthermore, the global economic and political factors have a great impact on food security and nutrition in the region. According to the Food and Agriculture Organization (FAO), the sharp rise in global food prices between 2021 and 2022 due to disrupted supply chains and rising fuel costs severely impacted food access in the region, which is heavily reliant on importation and currency depreciation. Inflation, driven by global economic instability, erodes purchasing power, making food less affordable and pushing more people into hunger and malnutrition. Additionally, Ukraine has significantly impacted global grain supplies, with MENA countries, which rely heavily on wheat imports from Russia and Ukraine, facing severe shortages and price hikes [105]. The prevalence of the population in IPC Phase 3 or above increased from 13% in October 2021 to 24% by September 2022, driven by a deteriorating economy, poor harvests, and conflict [29]. Between October 2023 and February 2024, 17.7 million people in Sudan, or 37% of the population, faced high levels of acute food insecurity (IPC Phase 3 or above), with 4.9 million in IPC Phase 4 (Emergency). The conflict in Sudan has severely jeopardized food security and healthcare access, displacing families and exacerbating the crisis. The estimated number of acutely malnourished individuals in Sudan in 2024 is 4.86 million, including 3.66 million children under 5 and 1.2 million pregnant and lactating women, marking a 22% increase within a year [106].

The latest IPC projection conducted in Sudan showed that **over half of the population** (25.6 M people) faced crisis or worse conditions (IPC Phase 3 or above) between June and September 2024, coinciding with the lean season. This includes 755,000 people in catastrophe (IPC Phase 5) in 10 states including Greater Darfur (all five states), South and North Kordofan, Blue Nile, Al Jazirah, and Khartoum. Another 8.5 M (18 percent of the population) face emergency (IPC Phase 4). There is a risk of famine in 14 areas (five localities and nine clusters of Internally Displaced Persons (IDPs) and refugees in Greater Darfur, Greater Kordofan, Al Jazirah states and some hotspots in Khartoum) if the conflict escalates further, including through increased mobilization of local militias [107].

Although key drivers affecting food security are common in most countries, the effect is influenced by their unique socio-political contexts, economic conditions, and environmental factors, for example. Yemen and Afghanistan face severe food insecurity driven by conflict and economic challenges, but the specific drivers and their impacts vary significantly due to the differing contexts of each country; both countries are affected by conflict and Yemen’s situation is characterized by ongoing civil war and economic warfare, whereas Afghanistan’s challenges stem from political instability following a regime change. Yemen faces economic warfare and high food prices, while Afghanistan struggles with broader economic collapse and inflation. Both countries rely on humanitarian assistance, and recent cuts in assistance, particularly in areas controlled by the Sana’a-based authorities in Yemen, have left millions vulnerable. The cessation of aid has resulted in increased food consumption gaps, pushing many households into crisis (IPC Phase 3) or emergency (IPC Phase 4) levels of food insecurity, while in Afghanistan, the political situation has complicated aid delivery, leading to inconsistent support for those in need [108,109].

In the Gaza Strip, occupied Palestine, food security and malnutrition levels have worsened since the onset of recent hostilities. An IPC analysis in 2023 showed that 93% of the population in Gaza was in IPC Phase 3 or above, which escalated to 100% in 2024. The percentage of households experiencing famine increased from 17% in 2023 to 50% in 2024. Before the recent conflict, wasting among children under five was rare, with only 0.8% affected. However, in Northern Gaza, the wasting rate among children under two surged to 15.6%, representing an unprecedented global decline in nutritional status within three months [110].

Similarly, in sub-Saharan Africa, studies have also shown the impact of conflict on food security. An analysis using household-level data from Malawi and Ethiopia revealed a direct relationship between violent conflicts and food security [111]. A community-based cross-sectional study in Tigray, Ethiopia, found that armed conflict significantly exacerbated household food insecurity and hunger [112].

Natural disasters, particularly climate-related events, also play a crucial role in affecting food security. For example, floods in Pakistan in 2022 led to a significant deterioration in food security, with 43% of households classified in IPC Phase 3 or above. The flooding caused livestock losses and negatively impacted food production, availability, and livelihood opportunities [113]. Similar patterns were observed in Afghanistan, where a combination of factors, including widespread humanitarian assistance, improved cereal harvests, and a strengthening currency, led to the lowest level of acute food insecurity since 2017. However, despite these improvements, malnutrition rates remain alarmingly high, reflecting the challenges of maintaining food security in conflict-affected regions [39]. The impact of natural disasters on food security is also evident in Southeast Nigeria, where floods increased the number of food-insecure households to 92.8%. The regression coefficient of −0.798 indicates a strong negative effect of flooding on household food security [113].

Scarcity of water and drought also affects food security and livelihood, Somalia and Sudan are highly vulnerable to climate change, particularly due to frequent droughts. In Somalia, recurring droughts have severely affected food security, leading to widespread famine and displacement, as seen during the 2017 drought, which impacted over 6 million people. Sudan faces both droughts and seasonal floods, with the 2020 floods displacing nearly 830,000 people and damaging critical infrastructure. Yemen is vulnerable to water scarcity and faces erratic rainfall patterns that lead to flash floods, particularly in coastal areas. The 2019 and 2020 floods displaced tens of thousands of Yemenis and worsened cholera outbreaks due to poor sanitation. Lebanon, on the other hand, contends with infrastructure fragility, especially after the 2020 Beirut explosion, which compounded the country’s vulnerability to climate impacts such as heatwaves and water scarcity. Meanwhile, Pakistan faces climate challenges, including extreme heatwaves and glacial melting, which increase the risk of glacial lake outburst floods (GLOFs) in the north [114].

Disease outbreaks, particularly the COVID-19 pandemic, have also emerged as critical drivers of food insecurity and malnutrition. The pandemic severely affected health, the economy, and access to health services, especially in low-income countries with fragile health systems. The COVID-19 pandemic has significantly exacerbated pre-existing vulnerabilities in health systems and nutrition supply chains across Eastern Mediterranean region (EMRO) countries. The negative impact was created due to several factors. Due to the diversion of health resources toward COVID-19 response efforts, resulting in reduced access to essential health services, many EMRO countries faced budget cuts and reduced health expenditures, which hindered their ability to maintain and improve health services. The pandemic placed immense pressure on healthcare workers, leading to burnout and shortages. The direct impact on nutrition was by affecting the distribution of food supply and treatments due to the lockdown and movement limitation. The pandemic led to an increase in food prices and a decrease in the diversity of food, especially in low-income countries; this, in turn, affected the level of malnutrition and food insecurity [115]. In India, a longitudinal study showed a sharp increase in food insecurity during the pandemic, with the prevalence of any food insecurity rising from 21% to 80% within six months [116]. The pandemic’s effects on food insecurity were reflected in malnutrition rates, as seen in Yemen, where 60% of households were in IPC Phase 3 or above, with a GAM rate of 22.1%. The continuity of conflict, economic crisis, and instability of humanitarian assistance in Yemen exacerbated food insecurity and malnutrition [117].

In Syria, the protracted conflict, economic crisis, and natural disasters, such as earthquakes, have worsened food insecurity and malnutrition. The GAM rate in Syria increased from nearly 1% in 2020 to 4.7% in 2022 and continued to rise in 2023. The ongoing conflict and economic hardship have forced millions into poverty, severely impacting children’s diets and nutritional status [100].

Even in countries where food security has improved, malnutrition remains a persistent issue. In Somalia, despite a decline in food insecurity from 45% to 29% in 2023 due to improved harvests and better rainfall, malnutrition rates continued to rise. Factors such as limited access to health and nutrition services, high morbidity, and insufficient humanitarian funds have contributed to the worsening nutrition status in the community [117].

Malnutrition is a chronic problem in developing and low-income countries, particularly in the Eastern Mediterranean region, where various forms of malnutrition coexist. This not only affects the health of the community but also hinders development, economic growth, and overall quality of life. The progress toward global nutrition targets set for 2025 varies across countries, depending on the specific drivers and challenges they face. For instance, Afghanistan has made progress in increasing exclusive breastfeeding rates and reducing child overweight rates; the combination of increased awareness, cultural shifts, supportive policies, and effective monitoring has enabled Afghanistan to make significant strides toward achieving its breastfeeding targets [118]. However, data on low birth weight are lacking in most countries, except Lebanon and Palestine, highlighting a gap in addressing maternal and child health issues that could prevent future health and nutrition hazards. The progress and reached values of global nutrition target indicators for the 10 countries were due to commitment from governmental and non-governmental organizations to include strategies and policies to enhance progress to reach nutrition targets. The combination of strong governmental policies, community engagement, improved healthcare access, food security initiatives, and international support has enabled countries like Iraq, Djibouti, Palestine, and Lebanon to make significant progress toward reducing stunting. Continued efforts in these areas are essential for sustaining and further enhancing nutrition outcomes for children [118]. Although some countries registered progress in some targets, like Afghanistan in reaching the targeted goal for exclusive breastfeeding and decreasing the rates of overweight in children, other countries are far from reaching their own goals. All the countries except Lebanon and Palestine lack data related to low birth weight either a description of the current situation of the country or the target to be reached. Low birth weight does not reflect only the health of the delivered baby; it reflects the health status of his mother during pregnancy. Maternal malnutrition and health problems could be factors leading to low birth weight, and it is highly important to be aware of the size of such problems to prevent future health and nutrition hazards for low-birth-weight babies.

Countries facing complex emergencies encounter numerous challenges that impact nutrition and health outcomes. According to the WHO’s Strategy for the Eastern Mediterranean region 2020–2030, these challenges include the following: (1) governance—weak institutional capacity of ministries of health in post-conflict phase in policy/planning; (2) financing—limited capacity to efficiently utilize public funds, revitalize social protection schemes; (3) health workforce—inadequate policies to attract emigrants home, absent human resources plan for rebuilding appropriate workforce, need to revitalize closed or poorly functioning institutions, over-reliance on expatriate workforce, limited ministry of health capacity to coordinate donors and ensure aid effectiveness, and lack of trained ministerial staff; (4) service provision—ineffective primary care and hospital-based services for handling emergencies, inappropriate balance between health services provided by public sector and by non-governmental organizations; (5) health information—weak information systems; and (6) health technologies—lack of fast-track mechanisms for procurement and regulation of technologies for countries in complex emergencies [119].

Due to the previous challenges in countries of emergency various organizations have implemented interventions to address these challenges and mitigate food insecurity and malnutrition.

Cash and voucher programs provided by the WFP, which allow beneficiaries to purchase food locally, not only address immediate food needs but also support local economies. In Yemen, for example, the WFP has implemented cash-based transfers to help families buy food, which is crucial given that around 83% of the population faces food insecurity [120].

From another side, organizations that work with refugees focus on providing food security for refugees through various means, including cash assistance and food vouchers. In Lebanon, for example, the UNHCR has reported that half of Syrian refugee households were food-insecure in 2020, prompting the agency to enhance its support through targeted food assistance programs [121]. Organizations concentrating on agriculture like the Food Agriculture Organization (FAO) promote urban agriculture and community gardens as sustainable solutions to food insecurity. Initiatives that encourage refugees and host communities to engage in urban farming were applied, which helped in improving food access and nutritional outcomes [121,122]. In conflict-affected areas, some humanitarian agencies find challenges in reaching targeted populations; the ICRC works in conflict-affected areas to provide food assistance. In Syria, for example, the ICRC has distributed food parcels and cash assistance to families in need, aiming to alleviate the impact of ongoing conflict on food security. UNICEF focuses on combating malnutrition, particularly among children and pregnant women. It implements targeted nutrition programs that include supplementary feeding and nutrition education.

Funds and support from stakeholders continue to decrease and mitigate food insecurity and malnutrition. The World Bank has funded projects aimed at restoring agricultural production and improving food security in affected countries. Local non-governmental organizations also work in the field to provide all the support needed [120].

This study faced significant limitations due to the availability and reliability of data. The analysis, which focused on five years from 2020 to 2024, was hindered by the lack of consistent and accurate data on malnutrition rates and food security. For example, in Syria and Iraq, food security analyses were not conducted during the study period, and there was a lack of data on malnutrition in children under five in Iraq. This created an unclear picture of the nutrition and food security status in these countries. The reliance on outdated data further posed challenges, as it may not accurately reflect current conditions. Additionally, conflicting data from different sources complicated the analysis and interpretation of the findings.

## 5. Conclusions and Recommendations

The study concludes that food security and malnutrition in emergency-affected countries are influenced by a combination of factors, including conflict, political instability, natural disasters, and socioeconomic conditions. The disruption of agricultural activities, displacement of populations, and collapse of infrastructure exacerbate these issues, leading to widespread food insecurity and malnutrition. The study also highlights the impact of climate change and natural disasters on agricultural productivity, as well as the ongoing effects of the COVID-19 pandemic on food security and health systems in low-income countries.

### To Address These Challenges, the Study Recommends

A multisectoral approach for coordinated efforts to combat food insecurity and malnutrition is exemplified by the SUN (Scaling Up Nutrition) movement, which includes countries like Afghanistan, Djibouti, Pakistan, Somalia, Sudan, and Yemen. This integrated approach is highly important in countries where the concept of work coordination and cooperation between different sectors is weak, UN organizations emphasize the importance of integrated approaches that combine food security with health, education, and social protection. This holistic strategy ensures that interventions are more effective and sustainable, addressing the multifaceted nature of food insecurity and malnutrition, and includes the following:

Prioritizing conflict resolution and peacebuilding to create a stable environment for sustainable food production.

Adopting climate-smart agriculture and investing in resilient infrastructure to mitigate climate impacts.

Integrating short-term emergency responses with long-term development strategies to achieve sustainable development and global nutrition goals.

Strengthening health and nutrition information systems for accurate and up-to-date data to guide stakeholders and humanitarian efforts.

Addressing factors like governance quality, international sanctions, access to global markets, and external aid responses is essential for improving food security and ensuring that vulnerable populations have access to adequate nutrition.

## Figures and Tables

**Table 1:** Last annual projections for IPC ANALYSIS, 5 phases (Phase 1–Phase 5) [9,10,11,12,13,14,15,16,17,18,19,20,21,22,23,24,25,26,27,28,29,30,31,32,33,34].

Country	2020					2021					2022					2023					2024				
	1	2	3	4	5	1	2	3	4	5	1	2	3	4	5	1	2	3	4	5	1	2	3	4	5
Afghanistan	24%	34%	28%	14%	0%	33%	38%	22%	7%	0%	20%	35%	31%	14%	0%	31%	40%	24%	5%	0%	28%	37%	28%	8%	0%
Djibouti	60%	26%	12%	2%	0%	48%	35%	15%	2%	0%	49%	35%	15%	1%	0%	42%	34%	16%	8%	0%	No Analysis				
Gaza	No analysis					No analysis					No analysis					0%	7%	34%	42%	17%	0%	0%	12%	38%	50%
Lebanon	No analysis					No analysis					18%	45%	31%	6%	0%	18%	40%	35%	7%	0%	No Analysis				
Pakistan	46%	29%	17%	7%	0%	40%	38%	17%	6%	0%	25%	32%	30%	13%	0%	33%	38%	23%	6%	0%	31%	36%	26%	6%	0%
Somalia	66%	23%	9%	2%	0%	65%	21%	12%	2%	0%	40%	19%	26%	13%	2%	40%	35%	19%	6%	0%	50%	32%	14%	4%	0%
Sudan	51%	35%	13%	2%	0%	55%	32%	10%	3%	0%	39%	37%	18%	6%	0%	26%	37%	27%	10%	0%	26%	37%	27%	10%	0%
Yemen	22%	33%	33%	12%	0%	17%	29%	37%	17%	0%	17%	23%	37%	23%	0%	27%	32%	29%	12%	0%	27%	28%	32%	13%	0%

**Table 2:** Phase 3 and above, 2020–2024 (summarized from Table 1).

**Phase 3 + 4 + 5**	**Country**	**2020**	**2021**	**2022**	**2023**	**2024**
	Afghanistan	42%	47%	45%	29%	36%
	Djibouti	14%	17%	16%	24%	NA
	Gaza	NA	NA	NA	93%	100%
	Lebanon	NA	NA	37%	42%	NA
	Pakistan	24%	23%	43%	29%	32%
	Somalia	11%	14%	41%	25%	18%
	Sudan	15%	13%	24%	37%	37%
	Yemen	45%	54%	60%	41%	45%

**Table 3:** Number of malnourished children under 5 years in 4 years [29,35,36,37,38,39,40,41,42,43,44,45,46,47,48,49,50,51,52,53,54,55,56,57,58,59,60,61,62,63,64,65,66,67,68,69,70,71,72,73,74,75,76,77,78,79,80,81,82,83,84,85,86,87,88,89,90,91,92,93,94,95,96,97,98,99,100,101,102].

	2020				2021				2022				2023			
Country Name	Children Under 5	SAM #	MAM #	GAM #	Children Under 5	SAM #	MAM #	GAM #	Children Under 5	SAM #	MAM #	GAM #	Children Under 5	SAM #	MAM #	GAM #
Afghanistan	6,375,097	NA	NA	NA	6,490,554	NA	NA	3,200,000	6,610,100	1,080,000	2,800,000	3,880,000	6,726,170	NA	NA	4,000,000
Djibouti	115,819	NA	NA	NA	116,373	NA	NA	NA	116,906	NA	NA	NA	117,412	5500	27,500	33,000
Iraq	NA	NA	NA	NA	NA	NA	NA	NA	NA	NA	NA	NA	NA	NA	NA	NA
Pakistan/SINDH 2022 DATA	29,433,002	NA	NA	NA	29,604,479	NA	NA	NA	29,798,188	126,090	510,160	636,250	30,015,901	600,000.0	1,500,000.0	2,100,000
Palestine	NA	NA	NA	NA	NA	NA	NA	NA	NA	NA	NA	NA	NA	NA	NA	NA
Lebanon	472,518	NA	NA	NA	445,136	NA	NA	NA	419,603	NA	NA	NA	395,739	NA	NA	NA
Somalia	1,915,790	178,000	830,000	1,008,000	3,200,131	162,000	800,000	962,000	3,27,7528	295,515	986,000	1,200,000	3,356,725	500,000	1,300,000	1,800,000
Sudan	6,991,112	522,000	2,200,000	2,722,000	7,083,969	570,000	2,430,000	3,000,000	7,192,566	560,000	2,200,000	2,760,000	7,289,083	611,000	2,400,000	3,000,000
Syria	1,835,971	33,558	103,592	137,000	1,892,447	NA	NA	MORE THAN 90,000	1,990,679	51,000	134,000	245,000	2,132,504	75,726	287,830	363,556
Yemen	4,682,245	400,000	1,630,000	1,960,000	4,714,059	395,195	1,860,000	2,250,000	4,760,445	538,000	1,662,000	2,200,000	4,760,445	1,050,000	1,150,000	2,200,000

**Table 4:** Malnutrition rates [29,103,104,105,106,107,108].

	2020			2021			2022			2023		
Country Name	SAM %	MAM %	GAM %	SAM %	MAM %	GAM %	SAM %	MAM %	GAM %	SAM %	MAM %	GAM %
Afghanistan	4.4%	12.6%	17.0%	4.4%	12.6%	17.0%	2.8%	11.3%	14.1%	1.2%	2.5%	3.7%
Iraq	3.0%	NA	NA	NA	NA	NA	NA	NA	NA	NA	NA	NA
Djibouti	NA	NA	10.3%	NA	NA	NA	4.4%	9.0%	13.4%	NA	NA	NA
Pakistan/Balochistan	4.30%	4.90%	9.20%	NA	NA	NA	7.4%	13.8%	21.2%	NA	NA	NA
Pakistan/Kybar Pakhtun	3.70%	7.4%	11.10%	NA	NA	NA						
Pakistan/Sindh	5.10%	14.80%	19.90%	NA	NA	NA				NA	NA	NA
Palestine	0.60%	1.30%	1.90%	NA	NA	NA	NA	NA	NA	NA	NA	5–15%
Somalia	2.30%	11.50%	13.80%	1.8%	11.3%	13.1%			11.1%	NA	NA	15.9%
Sudan	3.7–4.9%		16–19%			14.1%				NA	NA	13.6%
Syria			0.9%	0.80%	3.90%	4.70%	1.70%	3.30%	5%	NA	NA	5–12%
Lebanon	NA	NA	NA	0.30%	1.50%	1.80%	NA	NA	NA	NA	NA	NA
Yemen	0.9–6.2% 2.1% median	5.7–20.9% 11.4% median	6.6–27.1% 13.3% median	NA	NA	7–31% the median 15%	5.2%	16.9%	22.1%	NA	NA	NA

**Table 5:** Targets and current rates for global nutrition target 2025 [103].

	Stunting		Wasting		Anemia		Low Birth Weight		Exclusive Breastfeeding		Overweight	
Country	Target	Reached	Target	Reached	Target	Reached	Target	Reached	Target	Reached	Target	Reached
Afghanistan	16.30%	33.10%	5%	5.1% (2018)	18.80%	42.6% (2019)	no data	no data	56.70%	57.5%	5.0%	3.7% (2022)
Iraq	8.20%	9.90%	5%	3.00%	14.90%	28.6% (2019)	no data	no data	45.10%	25.8% (2018)	9.50%	6.4% (2022)
Djibouti	17.00%	18.70%	5%	10%	15.50%	32.3% (2019)	no data	no data	38.68%	12.4% (2012)	1.30%	3.2% (2022)
Pakistan	24.60%	34%	5%	7.10%	21.40%	41.3% (2019)	no data	no data	62.60%	47.80%	4.60%	2.7% (2022)
Palestine	5.50%	7.50%	5%	9%	15.30%	31%	10.40%	6.90%	52.80%	38.9% (2020)	7.60%	8.3% (2022)
Somalia	11.40%	18%	5%	14.3% (2009)	22%	43.10%	no data	no data	38.90%	33.7% (2018)	3%	2.7% (2022)
Sudan	16.80%	36%	5%	16.3% (2014)	18.40%	36.50%	no data	no data	60.90%	54.6% (2014)	2.40%	2.7% (2022)
Syria	19.90%	25.40%	5%	11.5% (2010)	15.90%	32.8% (2019)	no data	no data	62%	28.4% (2019)	16.60%	11.7% (2022)
Lebanon	9.40%	7.40%	5%	1.4% (2021)	no data	no data	12.60%	8.50%	50%	No data	8.50%	8.3% (2022)
Yemen	24.50%	35.10%	5%	16.4% (2013)	30.80%	61.5% (2019)	no data	no data	35%	9.7% (2013)	2.40%	1.7% (2022)

**Table 6:** Key drivers affecting food insecurity and malnutrition [9,10,11,12,13,14,15,16,17,18,19,20,21,22,23,24,25,26,27,28,29,30,31,32,33,34].

Country	2020	2021	2022	2023
Afghanistan	○ COVID-19 ○ High food prices ○ conflict ○ IDPs ○ Floods	○ Conflict ○ Drought ○ Economic decline	○ The effect of lean season	○ Climate shock ○ High food prices ○ Displacement ○ Reduced income and employment
Djibouti	○ COVID-19 ○ Desert Locust ○ Floods	○ COVID-19 ○ Desert Locust ○ Floods	○ Political and economic challenges ○ (Ukraine war and conflict in Ethiopia) ○ Low rainfall and prolonged dry season. ○ Loss of jobs ○ Limited access to safe water.	○ Increase in food prices. ○ Death of animals due to diseases ○ Low level of agricultural practice ○ Low production of local food ○ Decrease access to employment.
Lebanon			○ Inflation ○ Limited job opportunities ○ Loss of purchasing power ○ Currency depreciation ○ Limited value of assistance ○ Phase out of subsidies	○ Inflation ○ Currency depreciation ○ Discontinuation of subsidies ○ Loss of purchasing power ○ Limited job opportunities ○ Political uncertainty
Pakistan	○ limited availability of water ○ Prolonged conflict affecting livestock. ○ High inflation ○ Distance of Markets ○ “High level of debt among households ○ WASH challenges. ○ inaccessibility to healthcare.	○ High food prices ○ COVID-19 ○ Locust infestation ○ Drought (Balochistan) ○ Flooding (Sindh)	○ High food prices ○ COVID-19 ○ Drought/dry spells ○ Conflict and displacement ○ Livestock disease or death	○ High food prices ○ Reduced food production ○ Climate shocks ○ “Reduced employment opportunities/ ○ income” ○ Livestock disease and mortality
Somalia	○ Flooding ○ Desert Locust ○ COVID-19	○ Conflict ○ Erratic rainfall ○ Flooding ○ COVID-19	○ Prolonged drought ○ High food prices ○ Conflict	○ Conflict ○ Erratic rain ○ Diseases Flooding ○ Drought ○ High food prices ○ Disease and poor health access
Sudan	○ COVID-19 ○ Economic decline and inflation ○ Conflict	○ Economic decline/inflation ○ Floods ○ Conflict/displacement	○ Economic decline/inflation ○ Conflict ○ Poor harvest ○ The conflict in Ukraine	○ Conflict and insecurity ○ High food prices and Macroeconomic crisis ○ Climate shock and hazards ○ Low agriculture production
Yemen	○ Conflict ○ Economic decline ○ Low humanitarian food assistance ○ Reduced essential services ○ Morbidity ○ Conflicts ○ COVID-19	○ Conflict ○ Economic shock ○ Reduced foreign reserves.	○ Conflict ○ Economic decline ○ Climate change ○ High food and fuel prices	○ Conflict ○ Economic decline ○ Reduced access to essential services ○ Improved humanitarian food assistance
Palestine (Gaza Strip)				○ Hostilities ○ Access to services ○ WASH challenges
